# Addressing methodological and ethical issues in practicing health economic evaluation in China

**DOI:** 10.7189/jogh.10.020322

**Published:** 2020-12

**Authors:** Shan Jiang, Zhuo Chen, Jing Wu, Xiao Zang, Yawen Jiang

**Affiliations:** 1 School of Population and Public Health, University of British Columbia, Vancouver, British Columbia, Canada; 2 Department of Health Policy and Management, College of Public Health, University of Georgia, Athens, Georgia, USA; 3 School of Economics, Faculty of Humanities and Social Sciences, University of Nottingham Ningbo China, Ningbo, China; 4 School of Pharmaceutical Science and Technology, Tianjin University, Tianjin, China; 5 Department of Epidemiology, School of Public Health, Brown University, Providence, Rhode Island, USA; 6 School of Public Health (Shenzhen), Sun Yat-sen University, Shenzhen, Guangdong, China

Health economic evaluations provide guidance for allocating resources and improving health outcomes. In low- and middle-income countries with limited health resources such as China, economic evaluation should have a more significant role than it does. However, several practical issues may hamper the development of economic evaluations in China, including cost inventory, measurement of health outcomes, thresholds for willingness-to-pay, validity and ethics of economic modeling, and the capacity of fast evaluation when public health crises emerge. Stakeholders of the health care sector should collaborate closely to address the challenges and to deliver sound economic evaluations.

Through the application of analytic frameworks such as cost-effectiveness analysis and cost-utility analysis [1], health economic evaluation generates data-driven information to assist decision-making and resource allocation in health and health care. Widely used in high-income countries, economic evaluation provides evidence that enables health professionals and policy-makers to improve health outcomes [1]. In countries where the health care budget per capita is lower than that in high-income countries, economic evaluation should have a more significant role than it has, because the marginal return of health care budget should be higher than that in high-income countries. In 2018, China’s per capita health care expenditure was US$ 688, which is 6.5%, 13.8%, and 16.9% of the per capita expenditures in the US, Canada, and UK, respectively [2]. The numbers suggest the scarcity of health care resources in China and the necessity of developing health economic evaluation. As such, we aim to identify methodological and ethical issues associated with the practice of health economic evaluation in China that are in urgent need to be addressed.

There are several practical issues that stakeholders, such as policy-makers, physicians, and health economists, need to consider. First, a guide on cost inventory from different perspectives is overdue. The perspective is the point of view adopted by economic evaluations when deciding what types of costs and health benefits to be included. The most common ones are societal, health care system, and payer perspectives. The health care system perspective considers a broader range of costs and benefits than the payer perspective. The societal perspective is the broadest among the three, which reflects a full range of social opportunity costs that are directly or indirectly associated with the interventions of interest. In countries with universal health insurance, guidelines usually recommend the use of health care system perspective and encourage the use of societal perspective in addition to the base case analysis from the health care system perspective [3,4]. Because China has a *de facto* universal health insurance coverage, the Chinese guidelines have made similar recommendations (**Table 1**) [5]. However, it is urgent for Chinese stakeholders to discuss and clarify the types of costs that should be included from the health care system or societal perspective, which has not been made explicit in current guidelines.

**Table 1:** Comparison of health economic evaluation methods recommended in guidelines (China, UK, Canada, and Germany)

Methodological issue	China	UK	Canada	Germany
HTA agency	Not established	NICE	CADTH	IQWiG
Analysis method	CUA and CEA	CUA and CEA	CUA and CEA	CBA but also CUA and CEA (not standard practice)
Perspective	Healthcare system and societal	Payer (NHS) or societal if justified	Healthcare system and societal if justified	Usually statutory health insurance
Types of costs	Not explicitly specified	Direct medical, social services if justified	Direct medical	Depending on perspective: direct medical, and informal costs
Productivity loss	Recommended	Not recommended	Not recommended	Not necessary in base case. Consider if societal perspective
Preferred outcome measure	QALY	QALY (cost per life year gained, if CEA)	QALY	Medical outcomes
Utility score	Not available for many health conditions	Utility score from general population, by direct (eg, TTO, SG), indirect (EQ-5D), or systematic review	Utility score from general population, by direct (eg, TTO, SG), indirect (EQ-5D), or systematic review	Utility scores from patients, direct (eg, TTO, SG), indirect (specific PROM instruments)
WTP threshold	Not specified.	₤20,000-₤30 000 per QALY; empirically ₤12 936 per QALY	Not specified. CA$20 000, CA$50 000, and CA$100 000 are commonly used in research	Efficiency frontier (Institute’s own approach)
Cost-sharing level	High	No payment for both health care services and prescriptions	No payment for health care services. Pay the full cost of prescriptions if not covered by private insurance	No payment for health care services. Pay a small proportion of prescriptions costs (about 10%)

CADTH – Canadian Agency for Drugs and Technologies in Health, CUA – cost-utility analysis, CEA – cost-effectiveness analysis, CBA - cost-benefit analysis, HTA – health technology assessment. IQWiG – Institute for Quality and Efficiency in Health Care, NHS – National Health Service in UK, NICE – National Institute for Health and Care Excellence, PROM – patient-reported outcome measures, QALY – quality-adjusted life-year gained, SG – standard gamble, TTO – time trade-off, WTP – willingness-to-pay

Additionally, the inclusion of productivity loss quality-adjusted life years (QALYs) would result in double counting [6]. In practice, quantifying patients’ productivity loss may take substantial data collection effort to produce what seems to be controversial outcomes. It is also difficult to quantify the impact of a patient’s sickness on caregivers’ productivity when the caregivers are family members. While guidelines in other countries have dismissed productivity loss [4], the Chinese guidelines encourage the inclusion of productivity loss (**Table 1**) [5]. Considering the theoretical controversies, this issue is worth a revisit.

Second, stakeholders need to consider the measures for health outcomes and associated instruments. Utility-based measures are increasingly used in economic evaluations, among which QALY is one of the most popular metrics [1]. QALY refers to the life-years gained from the intervention of interest, adjusted by the quality of life [1]. QALY is not universally accepted by health technology assessment (HTA) agencies in different countries because the concept of QALY needs to be put into the context of a country. For example, the agencies in Australia and Canada encourage the application of QALY in economic evaluation [3,4], while the German HTA agency relies mostly on a series of clinical indicators [7]. Though the Chinese guidelines recommend the use of QALY and health economists are increasingly using QALY in practice [5], the Chinese HTA agency does not require the use of QALY as an outcome measure. We expect the agency to make recommendations on this issue with explicit reasoning for the appropriateness of QALY in the Chinese context.

**Figure:**
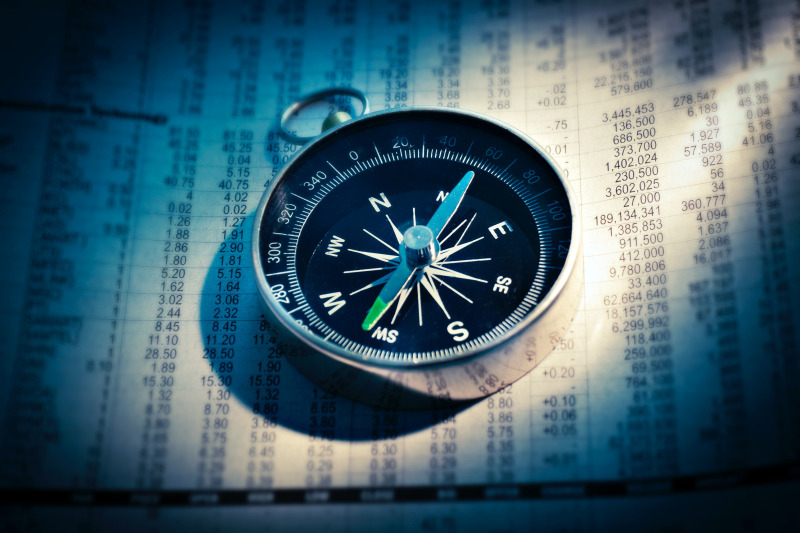
Photo: https://unsplash.com/photos/uCMKx2H1Y38.

If QALY is recommended, stakeholders should consider the instruments for health utility measurement of different health conditions. Generic and specific instruments from other countries are widely applied in China, such as EQ-5D, SF-6D, and EORTC for cancer patients. However, the validity and reliability of these instruments were questioned when adapted in China, and additionally, many health conditions have no utility values for economic evaluation (**Table 1**). It would be a long-term effort in China to facilitate health utility measurement, and possible solutions may include developing new instruments for Chinese population, mapping the non-preference-based measures to derive utilities, and modifying the practice guidelines of the established instruments in China.

Third, the threshold for willingness-to-pay (WTP) is a crucial issue for decision-making but remains unclear to the research community. Interventions with costs per QALY under the threshold will be considered for coverage by the payer. Thresholds used can be loosely divided into three groups, one, twice, and three times the GDP per capita. However, there is no single best approach to determine a threshold, and much less likely a threshold that fits all situations. Possible solutions may include: 1) identifying an implicit cut-off by investigating the national reimbursement lists of medications in the past three years, and 2) retrieving the medical records and costs of a representative cohort of patients by disease and using regression models to estimate the real-world costs for different diseases. These steps may provide prior information about the WTP thresholds for different drugs and health conditions. Additionally, the thresholds depend endogenously on the cost-sharing level,which determines high- or low-risk individuals to use the treatment. The latter affects the efficiency frontier and the cost-effective threshold in turn. It is not a major concern in countries with negligible cost-sharing, but causes concerns in China where the cost-sharing is significant (**Table 1**) [8].

Fourth, the use of health economic models calls for immediate attention. A health economic model is a mathematical process that simulates disease transmission, progression, diagnosis, and outcome under various intervention strategies in a unified framework. They are particularly useful in extrapolating long-term outcomes that are unavailable, unobservable, or unethical to collect. Economic models are increasingly used in China, but the validity of models and the availability of valid parameters are of major concern. Specifically, it is problematic to apply models from other countries in China, as the clinical pathways are significantly different between countries. There is also a lack of epidemiological evidence on many health conditions in China. The concerns call for the gathering of epidemiological data and the development of models in the context of China. As the popular saying “garbage in garbage out” forcefully stated, invalid models and biased parameters will lead to wrong conclusions that may hamper allocative efficiency in the health care sector.

Fifth, the ethical standards of economic evaluation practice need to be established. Many European countries adopt a “submission” workflow, requiring the pharmaceutical manufacturers who seek reimbursement coverage of their products to submit the economic evaluation models by themselves. The Chinese health authority adopts a submission workflow as in Europe. Pharmaceutical manufacturers use their own professionals or outsource the work to consulting firms. The workflow does not necessarily lead to biased conclusions but surely causes concerns about potential conflicts of interest. The modeling by HTA agencies or non-profit third-parties would prevent conflict of interest. The development of ethical standards and guidelines for the practice of economic evaluation in China may assuage such concerns.

Sixth, the Chinese HTA agencies need to build up the capacity of fast evaluation when public health crises emerge, such as infectious disease outbreaks. The COVID-19 pandemic is a reminder of how disruptive an infectious disease outbreak could be, and how much we need fast evaluation capability as decision-making assistance on issues such as the cost-effectiveness of different preventive, diagnostic, and treatment strategies. It requires HTA agencies to collect data, develop models, and predict outcomes quickly. Lack of evidence on cost-effectiveness will lead to suboptimal allocation of the limited health resources when public health crises emerge and cause unintended consequences for the control of infectious diseases.

Seventh, it is critical to consider non-health outcomes. The non-health outcomes refer to those outcomes surrounding the health care services but not directly associated with QALY outcomes, such as false-positive rate and waiting time. The Second Panel on Cost Effectiveness in Health and Medicine noted that decision makers need a “quantification and valuation of all health and non-health effects of interventions” [9]. Canadian guidelines have specified the consideration of non-health outcomes [4]. Chinese health economists may consider the valuation of non-health outcomes, which frequently relies on stated preference methods such as discrete choice experiment and best-worst scaling.

The first step in solving a problem is recognizing there is one. We identify several issues that need to be addressed for the development of health economic evaluation in China. They include the cost inventory from health system and societal perspectives, measurement of health outcomes, thresholds of willingness-to-pay, validity and ethical standards, capacity of fast evaluation, and the use of non-health outcomes. Collaboration and coordinated efforts among the stakeholders are needed to resolve these issues and produce concrete deliverables to improve the practice of health economic evaluation in China.
